# Congenital Atresia of the Oesophagus: *Reports of Three Cases*

**Published:** 1950-04

**Authors:** Ronald Belsey

**Affiliations:** Surgeon in charge, Department of Thoracis Surgery, Frenchay Hospital


### CONGENITAL ATRESIA OF THE OESOPHAGUS

### *Reports of three cases*

BY

RONALD BELSEY, F.R.C.S.

*Surgeon in charge, Department of Thoracic Surgery, Frenchay Hospital.*

---

This condition is not rare, but until recently has been almost invariably fatal. The condition is now curable, but early diagnosis is essential, and surgical correction of the abnormality must be performed within the first two or three days of birth. The diagnosis should always be suspected if the following signs are present: frothy mucus about the nose and mouth; cyanosis during feeds, and regurgitation; abdominal distention on screaming. The obstruction can always be felt on passing an adult size rubber catheter into the upper oesophagus.

The abnormality most frequently encountered consists of absence of the upper third of the thoracic oesophagus; the cervical oesophagus ends blindly, and the lower part of the oesophagus communicates with the trachea at the bifurcation. Cases 1 and 2, described below, belong to this category. The cases were shown at the Clinical Meeting of the Society on November 9th. Other abnormalities have been described, sometimes in combination, but are rare, e.g.

(*a*) Complete absence of whole oesophagus.

(*b*) The oesophagus is a solid cord.

(*c*) A normal oesophagus is separated from the pharynx by a mere web.

The variation found in Case 3 is extremely rare.

**Case 1.** Baby O. was admitted to the Thoracic Unit on December 18th, 1948, at the age of two days. Birth weight 7 lb. 12 oz. There was continual discharge of mucus from mouth and nose since birth; difficulty in taking feeds, and distressed breathing; one attack of cyanosis: general condition fair: both lungs wet. The abdomen became distended on screaming. X-ray examination after injection of lipiodol into the upper oesophagus through a catheter revealed complete oesophageal atresia at the level of the first rib: the intestines were seen to be distended with air, revealing presence of a tracheo-oesophageal fistula.

Operation performed on day of admission: right transpleural thoracotomy, with closure of tracheo-oesophageal fistula and end-to-end anastomosis of the upper and lower oesophageal segments; convalescence uneventful; feeding by mouth commenced thirty-six hours after operation.

Barium swallow six weeks after operation shows a perfectly functioning anastomosis and a well developed lower oesophageal segment. The child has remained well ever since, is now thirteen months old and feeding and developing normally.

**Case 2.** Andrew B. was admitted to the Thoracic Unit on October 10th, 1949, at the age of three days: full term, normal delivery, birth weight 8½ lb. Since birth there had been regurgitation of feeds with cyanotic attacks; and discharge of mucus from mouth and nose between feeds: moist sounds in both lungs, general condition fairly good. One previous child had died of congenital heart disease at the age of two years. X-ray of chest revealed collapse of the right upper lobe. Injection of lipiodol into the upper oesophagus through a rubber catheter showed complete atresia of the oesophagus at the level of the second rib, and intestines distended with gas.

Operation was performed on same day: right transpleural thoracotomy, with closure of tracheo-oesophageal fistula, and end-to-end anastomosis between the upper and lower oesophageal segments. The operation was difficult owing to shortness of the upper segment : the lower segment was very narrow and under-developed. Convalescence uneventful: feeding by mouth twenty-four hours after operation. Barium swallow ten weeks after operation showed a perfectly functioning anastomosis and considerable development of the lower oesophageal segment: child now well and feeding normally.

**Case 3.** Robert B. was admitted to the Thoracic Unit on September 28th, 1949, at the age of two days. He had been born in Southmead Obstetric Unit; five weeks premature, birth weight 5 lb., hydramnios present. No feeds were given first day, but a lot of mucus was present in the naso-pharynx. On the second day two feeds were given and both returned immediately; the baby became very cyanosed after the second feed: moist sounds present in both lungs, abdomen not distended, general condition poor. Under X-rays lipiodol injected through a catheter into the upper oesophagus revealed complete atresia at the level of the first rib: no gas present in the intestines.

Operation was performed on day of admission—Right transpleural thoracotomy. No tracheo-oesophageal fistula was present and the lower oesophageal segment extended only one inch above the diaphragm: the upper segment extended down to the azygos vein. The lower segment was replaced in the abdomen and the chest closed. The upper segment was then brought up to the surface and a cervical oesophagostomy performed. The lower oesophageal segment was next brought out on to the abdominal wall to form a gastrostomy: convalescence uneventful. The child is now five months' old and developing normally; there is a continual discharge of saliva from the oesophagostomy. At a later date the stomach will be brought up through the left side of the chest and a cervical oesophago-gastrostomy performed.

